# Assessment of Coping Strategies and Their Associations With Health Related Quality of Life in Patients With Chronic Heart Failure: the Brief COPE Restructured

**DOI:** 10.14740/cr385w

**Published:** 2015-04-06

**Authors:** Catarina Nahlen Bose, Gunilla Bjorling, Magnus L. Elfstrom, Hans Persson, Fredrik Saboonchi

**Affiliations:** a The Swedish Red Cross University College, Stockholm, Sweden; b Karolinska Institutet Department of Clinical Sciences Danderyd Hospital, Stockholm, Sweden; c Malardalen University, Academy of Health, Care and Social Welfare, Eskilstuna/Vasteras, Sweden; d Karolinska Institutet, Department of Clinical Neuroscience, Division of Insurance Medicine, Stockholm, Sweden

**Keywords:** Heart failure, Factor analysis, Statistical, Adaption, Psychological, Quality of life, Self-report

## Abstract

**Background:**

Individuals with chronic heart failure (CHF) need to cope with both the physical limitations and the psychological impacts of the disease. Since some coping strategies are beneficial and others are linked to increased mortality and worse health-related quality of life (HRQoL), it is important to have a reliable and valid instrument to detect different coping styles. Brief COPE, a self-reporting questionnaire, has been previously used in the context of CHF. There is, however, currently a lack of consensus about the theoretical or empirical foundations for grouping the multiple coping strategies assessed by Brief COPE into higher order categories of coping. The main purpose of this study was to examine the structure of Brief COPE, founded on the higher order grouping of its subscales in order to establish an assessment model supported by theoretical considerations. Furthermore, the associations between these higher order categories of coping and HRQoL were examined to establish the predictive validity of the selected model in the context of CHF.

**Method:**

One hundred eighty-three patients diagnosed with CHF were recruited at a heart failure outpatient clinic or at a cardiac ward. Self-reported questionnaires were filled in to measure coping strategies and HRQoL. Confirmatory factor analyses were performed to investigate different hierarchical structures of Brief COPE found in the literature to assess coping strategies in patients with CHF. Regression analyses explored associations of aggregated coping strategies with HRQoL.

**Results:**

A four factorial structure of Brief COPE displayed the most adequate psychometric properties, consisting of problem focused coping, avoidant coping, socially supported coping and emotion focused coping. Avoidant coping was associated with worse HRQoL in CHF.

**Conclusions:**

This study provides support for a four-factor model of coping strategies in patients with CHF. This could facilitate assessment of coping both in clinical and research settings.

## Introduction

Chronic heart failure (CHF) is a syndrome characterized by shortness of breath, fatigue and peripheral edema. The prevalence is 2% in the general population [1] but rises sharply with age where 10% of the population aged ≥ 70 years have CHF [2]. Moreover, people with CHF experience worse health-related quality of life (HRQoL) compared to the general population [3]. Physical limitations and psychological distress impact the HRQoL for this patient group [4, 5]. In fact, having both CHF and depression predicts higher mortality rates [6] and readmission to hospital [7], as well as lower HRQoL [8].

Beyond the physical limitations, individuals with CHF also need to cope with the psychological impacts of the illness [9, 10]. Coping, i.e. the process of “constantly changing cognitive and behavioral efforts to manage specific external and/or internal demands” [11], is specifically significant in the context of CHF, as maladaptive coping strategies are linked to worse HRQoL [12]. Coping strategies have been viewed from two perspectives in research [13]: situational coping strategies, i.e. coping strategies used in a specific situation [11], and dispositional coping strategies, meaning coping strategies generally used by the individual [14]. The latter has been the target of most studies of coping and health outcomes in CHF [15]. Given that coping strategies have been linked to a number of health outcomes, i.e. anxiety and depression [16], clinical interventions in CHF [17] have been developed to enhance coping skills. Consequently, the assessment of coping strategies in CHF has gained significant importance in both clinical and research settings.

A large number of self-report instruments have over the years been developed to assess coping strategies, and have been used in populations suffering from CHF [16, 18-21]. Among these instruments are Ways of Coping Questionnaire (WCQ) [22], the Coping Inventory for Stressful Situations (CISS) [23], the Dealing with illness-R checklist [24], coping orientation to problems experienced (COPE) [25] and Brief COPE [26]. Conceptual accounts of coping, which classify coping efforts into different types, constitute the theoretical underpinning of these instruments.

COPE [25] is a multidimensional self-assessing instrument based on two theoretical models: Lazarus model about stress [11] and the model about behavioral self-regulation [27], and consists of 15 subscales each tapping a different coping strategy. At a higher level of theoretical hierarchy, these 15 coping strategies are assumed to be grouped into three different styles of coping, namely problem focused coping, emotion focused coping and maladaptive coping [25].

A short version of COPE, Brief COPE [26], was later introduced to address the difficulties associated with answering an extensive 60 items questionnaire. Brief COPE was the selected measure of coping strategies in the present study, as it has been used to assess dispositional coping strategies in various patient groups [28-31] and in the CHF population [21, 32-35]. Some practical, as well as conceptual, issues, i.e. several analyses, fragmentation of results and overlapping coping strategies, in the use of data from Brief COPE may arise due to the large number of subscales in this instrument.

To address such issues and in order to facilitate analysis of coping strategies in the CHF population, some studies have attempted to arrive at higher factors encompassing aggregates of several coping strategies. Based on either exploratory factor analysis, or principal component analysis, Brief COPE has been suggested to consist of two [32, 33] and three [34] factors. Higher order factors have also been proposed, based on the structure of the COPE [25, 36] where Brief COPE has been suggested to consist of four second order factors [21, 35].

Despite these attempts to restructure Brief COPE for use in the context of CHF, there is currently a lack of consensus about the theoretical or empirical foundations for grouping the multiple coping strategies into more overreaching categories of coping.

Since different styles of coping can affect health outcomes either positively or negatively in patients with CHF, it is important to have a reliable and valid instrument to assess coping styles. The aim of this study was to examine the structure of Brief COPE, founded on the higher order grouping of its subscales in a confirmatory approach, in order to establish an assessment model supported by theoretical considerations. Furthermore, we examined the associations between these higher order categories of coping with HRQoL in CHF in order to establish the predictive validity of the selected assessment model of Brief COPE.

## Method

### Participants

Patients (n = 183) with CHF were recruited during two time periods. In the first cohort, 80 patients were included consecutively during November 2007 and June 2008 at a nurse-led heart failure outpatient clinic or at a cardiac ward. In the second cohort, 103 patients were included consecutively during March 2011 and September 2013 and also recruited retrospectively from a waiting list, 2008 - March 2011, at a heart failure outpatient clinic. The participants met the following criteria: patients diagnosed with CHF hospitalized at a heart failure ward or at a nurse-led heart failure outpatient clinic at a hospital in mid-Sweden, were classified in New York Heart Association (NYHA) class II-IV, and were aged over 18 years. Exclusion criteria were cognitive dysfunction and/or life-threatening disease, such as cancer or primary organ failure, and not being able to understand the Swedish language. In the second cohort NYHA class IV was also an exclusion criterion. Patients who were accepted to join the study were administered the self-reported questionnaires together with a pre-stamped envelope. Participants also filled in their demographic data on a form. The clinical data were gathered from patient journals by the first author. The mean age was 71 years, the majority was men and 60% had been diagnosed with CHF for more than 1 year. The significant differences in the two cohorts concerned NYHA class (χ^2^ = 27.5, P < 0.001), duration of CHF (χ^2^ = 13.6, P < 0.01) and left ventricular ejection fraction (LVEF) (χ^2^ = 8.2, P < 0.05). Table 1 shows the demographic and clinical variables.

**Table 1:** Demographic and Clinical Data of the Total Sample, n = 183 (Cohort 1, n = 80 and Cohort 2, n = 103)

	Total sample		Cohort 1 (%)	Cohort 2 (%)
	n	%		
Age (mean ± SD)	71.3 ± 9.7 (range 39 - 94)		72.1 ± 10.5	70.7 ± 9.1
Sex (female)	54	29.5	27.5	31.1
Marital status				
Married/cohabitant	117	64.0	60.0	67.0
Single	66	36.0	40.0	33.0
Education				
Compulsory school	53	29.0	35.0	24.3
Upper secondary school	69	37.7	38.8	36.9
University	61	33.3	26.2	38.8
Occupation				
Working	35	19.1	18.8	18.4
Pensioner	140	76.5	77.5	75.7
Disability pensioner	5	2.7		5.8
Other	3	1.6	3.7	
NYHA class^a^				
Class II	126	68.9	48.75	84.5
Class III	55	30.1	48.75	15.5
Class IV	2	1.1	2.5	
Duration of heart failure				
Less than 6 months	48	26.2	18.8	32.0
6 months to 1 year	25	13.7	17.5	10.7
1 year to 2 years	30	16.4	11.2	20.4
2 years to 5 years	43	23.5	22.5	24.3
More than 5 years	37	20.2	30.0	12.6
Heart failure medication				
ACEi^b^	116	63.4	61.3	65.0
ARB^c^	61	33.3	35.0	32.0
Beta-blockers	178	97.3	96.3	98.1
Aldosterone receptor antagonist	88	48.1	53.8	43.7
Diuretics	148	80.9	92.5	70.9
Left ventricular ejection fraction (LVEF)				
Normal (LVEF > 50%)	27	14.8	16.2	13.6
Mildly reduced (LVEF 40-49%)	43	23.5	15.0	32.0
Moderately reduced (LVEF 30-39%)	68	37.2	35.0	38.8
Severely reduced (LVEF < 30%)	45	24.6	33.8	15.5

^a^NYHA class: New York Heart Association class. ^b^ACEi: Angiotensin Converting Enzyme inhibitor. ^c^ARB: Angiotensin II Receptor Blocker.

The study is confirmed by the Regional Ethics Review Board at Karolinska Institutet Stockholm, Sweden and all participants gave informed consent to participate.

### Measures

#### Brief COPE

Brief COPE consists of 28 items that measure 14 different coping strategies: active coping, planning, positive reframing, acceptance, humor, religion, using emotional support, using instrumental support, self-distraction, denial, venting, substance use, behavioral disengagement, and self-blame.

The main question was: What do you usually do when you are stressed by a problem? The coping strategies are described in statements such as: “I work or do other things in order not to think about the problem”. Each statement is graded on a four-point Likert scale: 1 = very seldom, 2 = fairly seldom, 3 = fairly often, 4 = very often. Each of the 14 coping strategies is indicated by two items. The Swedish version of Brief COPE has been psychometrically tested and proved adequate properties [37].

#### RAND 36-item Health Survey 1.0

The RAND 36-item Health Survey 1.0 [38] measures HRQoL including eight health concepts: physical functioning, role limitation due to physical health, role limitation due to emotional problems, energy/fatigue, emotional well-being, social functioning, pain and general health. Scoring is made by recoding the items between 0 and 100 where higher score indicates a better health condition. Then the items are averaged to create each scale and missing data are not taken into account, so the average represents the items actually responded in each scale. RAND 36 is also approached as comprising of a physical and a mental health composite [39]. One hundred and three patients filled in RAND 36 as it was implemented in the second cohort of the research.

### Statistical analysis

Statistical analyses were performed in SPSS, Amos version 22 and M-plus 7.1. At first Pearson correlation analysis between all the Brief COPE subscales were carried out and mean inter-correlation coefficient for each subscale was calculated.

Confirmatory Factor Analyses (CFA) with maximum likelihood estimation were performed on the different models of Brief COPE in the CHF population [21, 32-35] and on the original higher structure of COPE [25]. For evaluating the proposed measurement models, Chi-square test statistics were used. As χ^2^ statistics are overly sensitive to departure from multivariate normality and sample size, it may reject even well-fitted models [40]. Consequently, the following fit indices were further examined: Comparative Fit Index (CFI), Root Mean Squared Error of Approximation (RMSEA) and Standardized Root Mean square Residual (SRMR). For comparison between nested models the Likelihood Ratio Test (LRT, Δχ^2^) [41] was utilized. Modification Indices (MI) were inspected to examine theoretically justifiable modifications to improve fit of acceptable models.

Parceling method [42] was used to examine the higher order structure of Brief COPE. By parceling, each aggregate of a subscale is used as an indicator for the latent factors that are hypothesized to reflect the grouping of the coping strategies. This approach was due to the first order structure of Brief COPE not being identified, as the subscales were comprised of only two items each.

In order to account for the predictive validity of the higher order structure of Brief COPE, hierarchical linear regression analysis with measures of physical and mental health composites in the RAND 36-item Health Survey 1.0 [39] as dependent variables was performed. In the first block age, NYHA class and duration of CHF (dichotomized in < or ≥ 6 months) were entered and in the second block the higher order categories of coping strategies in Brief COPE were entered together with the significant predictors in the first block as independent variables.

### Models included in confirmatory factor analysis

1) The two-factor model presented in Bean et al [32]. 2) The two factor model presented in Eisenberg et al [33]. 3) The three-factor model suggested by Paukert et al [34]. 4) A four-factor model of Brief COPE shown by Perez et al [35] based on the factorial structure of COPE [25]. 5) A four-factor model of Brief COPE based on the original COPE [25] but with fewer omitted subscales than in the previous mentioned model by Perez et al [35]. 6) A four-factor structure of Brief COPE suggested by Nahlen et al [21] based on a model of COPE presented by Litman [36].

## Results

The mean inter-correlation coefficients between the subscales in Brief COPE varied between 0.07 and 0.28 (Table 2), indicating on average a weak association between the different subscales. Substance use and religion had the lowest mean inter-correlations with all the other subscales. Active coping and planning had the highest mean inter-correlation (0.58) followed by emotional support and instrumental support (0.56).

**Table 2:** Pearson Correlation Coefficient and Mean Inter-Correlation Coefficient of Subscales in Brief COPE

	1	2	3	4	5	6	7	8	9	10	11	12	13	14
1. Active coping														
2. Planning	0.58													
3. Positive reframing	0.43	0.48												
4. Acceptance	0.37	0.45	0.50											
5. Humor	0.28	0.32	0.38	0.28										
6. Religion	0.09	0.13	0.25	0.09	-0.01									
7. Emotional support	0.32	0.17	0.27	0.24	0.21	0.16								
8. Instrumental support	0.39	0.51	0.35	0.19	0.30	0.26	0.56							
9. Self-distraction	0.33	0.25	0.30	0.11	0.18	0.18	0.19	0.19						
10. Denial	-0.02	0.01	0.11	-0.08	0.01	0.05	0.06	0.10	0.38					
11. Venting	0.23	0.21	0.17	0.07	0.11	0.27	0.31	0.41	0.33	0.27				
12. Substance use	0.01	0.00	-0.04	-0.08	0.09	-0.07	0.00	0.08	0.11	0.07	0.09			
13. Behavioral diseng.	-0.15	-0.09	-0.01	-0.10	-0.07	0.05	-0.05	0.01	0.28	0.48	0.18	0.30		
14. Selfblame	0.26	0.40	0.39	0.18	0.16	0.25	0.16	0.35	0.36	0.18	0.48	0.04	0.26	
Mean inter-correlation coefficient	0.27	0.28	0.28	0.21	0.18	0.14	0.21	0.28	0.25	0.14	0.24	0.07	0.16	0.27

The six different factorial models of Brief COPE were analyzed with CFA. The two two-factor models and the three-factor model showed poor overall model fit indices as seen in Table 3 [21, 25, 32-35]. Increasing the number of factors into a four-structure model of Brief COPE resulted in overall better model fit values although the χ^2^ values remained significant.

**Table 3:** Model Fit Values on Confirmatory Factor Analysis (CFA) for Different Models of Brief COPE in Heart Failure Population. Calculated With M-plus

	χ^2^	df	CFI^b^	RMSEA^c^	90% CI^d^	SRMR^e^	Omitted subscales
Two-factor model, Bean et al [32]	279.78*	76	0.676	0.121	0.106 - 0.136	0.103	None
Two-factor model, Eisenberg et al [33]	224.59*	53	0.703	0.133	0.115 - 0.151	0.099	Humor, Religion
Three-factor model Paukert et al [34]	275.49*	63	0.643	0.136	0.120 - 0.152	0.114	Humor
Four-factor model, Perez-Garcia et al [35]	64.02*	17	0.878	0.123	0.092 - 0.156	0.070	Denial, humor, religion, self-distraction, substance use, venting
Four-factor model Carver et al (from COPE) [25]	53.18*	21	0.922	0.092	0.061 - 0.122	0.058	Humor, religion, self-blame, self-distraction, substance use
Four-factor model Nahlen and Saboonchi [21]	91.67*	49	0.911	0.069	0.049 - 0.091	0.063	Self-blame, self-distraction
Modified four-factor model Nahlen and Saboonchi^a^ [21]	70.43*	47	0.951	0.052	0.024 - 0.076	0.060	Self-blame, self-distraction

^a^Error variance for planning is correlated with the error variance for instrumental support and emotional support. *P < 0.001. ^b^CFI: Comparative Fit Index. ^c^RMSEA: Root Mean Squared Error of Approximation. ^d^CI: Confidence Interval. ^e^SRMR: Standardized Root Mean square Residual.

The four-factor model of Brief COPE presented by Perez et al [35] did not reach satisfactory fit statistics, whereas a proposed four-factorial structure of Brief COPE derived from the structure of the original COPE approached acceptable fit statistics with the exception of RMSEA [43].

**Figure 1:**
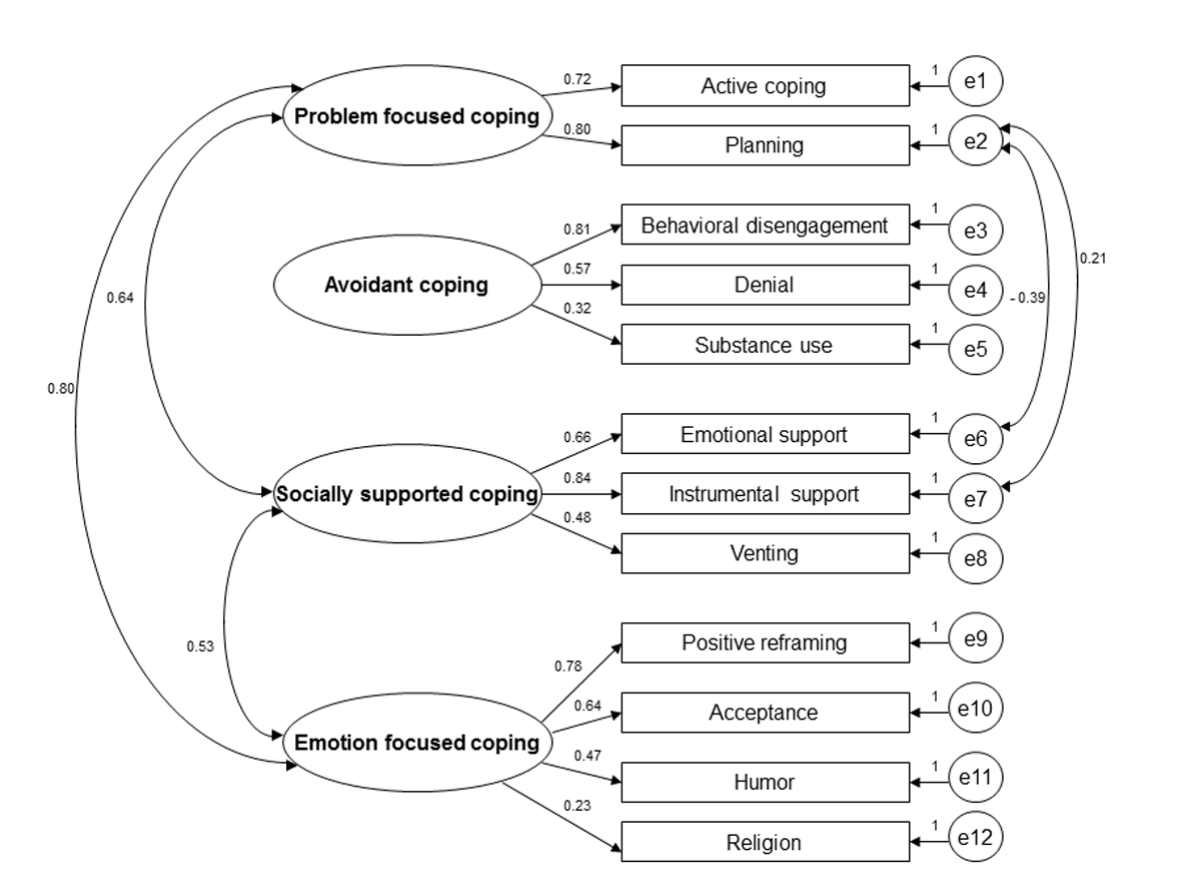
Modified four-factor model of Brief COPE. Parameter estimates are standardized coefficients. Non-significant correlations between the latent factors are not displayed in the model.

The four-factor model proposed by Nahlen et al [21] demonstrated fairly good model fit indices (Table 3). Examining MI for this model revealed that it was further improved if the error variance for planning, emotional support and instrumental support were allowed to co-vary (“modified four-factor model”, Table 3). The improvement of the fit of this model on basis of these modifications was further supported by the LRT (Δχ^2^ = 21.24, df = 2; P < 0.001). The model is displayed in Figure 1.

Mean and standard deviation for the four coping strategies are presented in Table 4 for the total sample, men and women and by median age. Cronbach’s alpha for the four factors of Brief COPE were as follows: problem focused coping, α = 0.78; avoidant coping, α = 0.51; socially supported coping, α = 0.62; emotion focused coping, α = 0.62.

**Table 4:** Mean and Standard Deviation of the Four Factors of Coping Strategies in Brief COPE for the Total Sample, Men, Women, Below and Over Median Age of 71 Years

	Problem focused coping (two subscales)	Avoidant coping (three subscales)	Socially supported coping (three subscales)	Emotion focused coping (four subscales)
Theoretical range	4 - 16	6 - 24	6 - 24	8 - 32
Man	10.7 ± 2.8	8.6 ± 2.5	12.9 ± 3.6	17.7 ± 4.4
Woman	9.8 ± 3.2	9.6 ± 3.1	12.5 ± 3.9	15.4 ± 3.5
Age < 71	10.9 ± 2.9	9.4 ± 2.8	13.4 ± 3.8	17.3 ± 4.2
Age ≥ 71	9.4 ± 3.0	8.2 ± 2.4	12.1 ± 3.5	16.7 ± 4.3
Total sample	10.5 ± 2.9	8.9 ± 2.7	12.8 ± 3.7	17.1 ± 4.3

The multiple hierarchical regression analyses with the physical and mental health composites of RAND 36 as dependent variables showed that avoidant coping and NYHA class significantly inversely predicted both physical and mental aspects of HRQoL (Table 5). The total explained variance of the multiple regression analysis of the physical health composite was 26%, and the corresponding explained variance for mental health composite was 40%.

**Table 5:** Hierarchical Linear Regression Analyses of Physical and Mental Health Composites in RAND 36 as Dependent Variables

	Standardized beta	*t*	R^2^
Dependent variable: physical health composite RAND 36			
Model 1			0.18
Age	0.31	3.06*	
NYHA class^a^ (NYHA III vs. NYHA II)	-0.30	-2.87*	
Duration of CHF^b^ (≥ 6 months vs. < 6 months)	0.11	1.10	
F(3) = 6.05, P = 0.001			
Model 2			0.26
Age	0.19	1.78	
NYHA class (NYHA III vs. NYHA II)	-0.31	-3.07*	
Problem focused coping	0.01	0.10	
Avoidant coping	-0.30	-2.79*	
Socially supported coping	-0.10	-0.92	
Emotion focused coping	-0.04	-0.31	
F(6) = 4.61, P < 0.001			
Dependent variable: mental health composite RAND 36			
Model 1			0.17
Age	0.33	3.27*	
NYHA class (NYHA III vs. NYHA II)	-0.29	-2.83*	
Duration of CHF (≥ 6 months vs. < 6 months)	-0.01	-0.12	
F(3) = 5.50, P = 0.002			
Model 2			0.40
Age	0.13	1.46	
NYHA class (NYHA III vs. NYHA II)	-0.26	-3.04*	
Problem focused coping	-0.01	-0.14	
Avoidant coping	-0.57	-6.31**	
Socially supported coping	-0.07	-0.75	
Emotion focused coping	-0.02	-0.16	
F(6) = 11.36, P < 0.001			

n = 103. *P < 0.01, **P < 0.001. ^a^NYHA class: New York Heart Association Class. ^b^Duration of Chronic Heart Failure.

## Discussion

The main purpose of the present study was to investigate a higher order grouping of subscales of Brief COPE used in the context of CHF. This study is unique in the sense of conducting a thorough investigation of different models of Brief COPE applied in research on patients with CHF. A four-factorial structure of Brief COPE displayed the most adequate psychometric properties in this study. The four factors consisted of problem focused coping, avoidant coping, socially supported coping and emotion focused coping. Among these categories of coping, avoidant coping was associated with worse HRQoL in CHF. These findings lend support to theoretical accounts of coping and its significance for health outcomes.

In Folkman and Lazarus seminal work, coping was conceptualized as two types: problem focused coping and emotion focused coping [22]. Problem focused coping is cognitive and behavioral efforts to reduce the stress by trying to solve the problem [11]. According to our results, problem focused coping consisted of active coping and planning, both of which may be viewed as strategies to approach a problem. The grouping of these strategies into problem focused coping also corresponds to Carver and Scheier’s original model of COPE [25]. The emotion focused coping factor in our study included positive reframing, acceptance, humor and religion. Emotion focused coping aims at reducing the stress by managing the emotions directed towards the stressor to regulate the emotional distress [11]. Conceptually, Lazarus and Folkman also include strategies generally viewed as maladaptive, e.g. behavioral disengagement and denial, in the definition of emotion focused coping. Our findings, however, separated those avoidant strategies from emotion focused coping in line with Carver et al [25]. In fact research has demonstrated that emotional approach is uncorrelated with avoidance [44].

Avoidant coping, in our study, was comprised of behavioral disengagement, denial and substance use. These strategies are characterized by avoiding thinking about the stressful situation and/or distract oneself from the stressor. The aim of these coping strategies is to protect oneself against being overwhelmed when encountering a very stressful situation [45]. Indeed, for most persons, CHF is an incurable condition with a poor prognosis [2]. Given the potential grandiosity of such a threat, avoidant coping may serve such a protective function. Avoidance, however, in long term is suggested to be dysfunctional [46]. An important finding in the present study pointed out avoidance coping to be independent from the other higher order grouping of coping strategies. This could imply that the patients employ avoidant coping style regardless of utilizing other coping efforts. A possible explanation may be the temporal aspects of coping [46] according to which different coping strategies may be used in different periods of time. For instance the patient might use avoidance coping in the initial phase and/or later on use avoidant coping in some situations and other coping strategies in other situations.

Seeking emotional support, seeking instrumental support and venting were shown to reflect the socially support coping factor in the present study. This grouping partially parallels the conceptual analysis of social support comprising four aspects: instrumental support, emotional support, tangible support and appraisal support [47]. Carvers et al’s [25] definition of seeking instrumental support includes seeking both the informational and tangible support. Seeking emotional support and venting may, in turn, aim at acquiring emotional and appraisal support [47]. Since the instrumental aspects of social support may benefit efforts to solve a problem or a stressful life situation, social support seeking may partly overlap with problem focused coping. Within the heart failure population social support has been shown to increase self-care behavior [48] which could imply better planning. Instrumental and tangible support is, by definition, directly linked to taking some form of action, whereas emotional support refers to providing love, empathy, care and trust [47]. Seeking emotional support, thus, may imply an effort to receive emotional comfort and soothing rather than planning to solve a problem at hand. Inspection of the items in instrumental support, e.g. “I get help and advice from other people”, and emotional support, e.g. “I get comfort and understanding from someone”, suggested viability of conceptual support for allowance of covariations of error terms of these coping strategies.

The results of our study revealed avoidant coping as a single factor to be adversely associated with both physical and mental health composites of HRQoL, meaning avoidant coping is related to worse HRQoL. Avoidant coping style has been studied in patients with CHF and has been shown to impact depressive symptoms [18], anxiety, fatigue [16] and predict increased mortality [49]. Our findings corroborate the maladaptive nature of avoidant coping in CHF, and provide evidence for predictive validity of our assessment model regarding this coping style. Our results indicate that avoidant coping may be an important factor to target in patients with CHF, for example via psychoeducational interventions.

The other factors, problem focused coping, emotion focused coping and socially supported coping, did not show any significant association with HRQoL in this study. Previous researches within the CHF population have found similar results [12], although seeking social support has been adversely associated with HRQoL [50]. With regard to other outcomes in emotional well-being, such as depression and affect, former studies have however found problem focused coping and single indicators for the emotion focused category to have a favorable association [18, 20, 21, 51]. Our findings in this regard, thus, are inconsistent with studies reporting direct associations between these coping strategies and emotional well-being. This inconsistency may be due to the use of other measures of coping in some of these studies [18], as well as to differences in the opted factorial structures in Brief COPE.

Functional status indicated to be adversely linked with HRQoL. This finding is consistent with previous research [4].

### Limitations

A limitation in this study is that the first order measurement model of Brief COPE with single items as indicators could not be identified due to the original structure of Brief COPE (i.e. two items for each subscale and a lack of specification of co-variances between the included coping strategies). Parceling, thus, was viewed as a viable solution to examine the higher order structure of Brief COPE. Another limitation was that the included models of Brief COPE in this study did not in all cases include the same indicators for the higher order factors. However, the indices for all but one of these models were poor rendering a model comparison unnecessary. Furthermore, the included subscales in the selected four-factor model of Brief COPE in this study were derived from Carver’s original construction of the COPE [26]. In our selected model, two subscales in Brief COPE, self-distraction and self-blame, were not included in the four-factor model based on the original structure of COPE. Although including these subscales in the avoidant coping factor seems conceptually appropriate, the negative associations between avoidant coping and HRQoL, despite excluding these subscales, support the predictive validity of our selected measurement model. The cohorts included in the present study also displayed some heterogeneity in regard to e.g. NYHA class and duration of CHF. The invariance of the selected assessment model in the present study needs to be established across subgroups of CHF patients in regard to relevant clinical parameters. Establishing measurement invariance across all possible clinical subgroups requires a substantially large sample size beyond the scope of the present study. However, this study provides a so called “parent” measurement model that can be used in future invariance testing of Brief COPE in this regard.

Finally and despite the overall acceptable fit, Cronbach’s alpha for all but problem focused coping were low. It may however be argued that, rather than a lack of reliability, low alpha may indicate constructs which encompass a heterogeneous spectra of related phenomena [52, 53] such as various different but conceptually related facets of coping.

### Conclusions

Valid and reliable measurement of coping strategies in patients with CHF is of great importance. This study provides an empirically sound and theoretical supported structure model of Brief COPE for measuring coping strategies in patients with CHF. This measurement model may facilitate the assessments compared to the model with 14 different subscales. Specifically, the model provides valuable information about avoidant coping which may be a clinically important target to identify in patients with CHF.
